# Providing Education and Training to Health Care Professionals to Address COVID-19 Health Disparities: Protocol for Implementation Project Using Reach, Effectiveness, Adoption, Implementation, and Maintenance Framework

**DOI:** 10.2196/60901

**Published:** 2025-05-16

**Authors:** Adati Tarfa, Nada Fadul, Erica Stohs, Jeffrey Wetherhold, Mahelet Kebede, Nuha Mirghani, Muhammad Salman Ashraf

**Affiliations:** 1 School of Medicine Yale University New Haven, CT United States; 2 Division of Infectious Diseases, Department of Internal Medicine University of Nebraska Medical Center Omaha, NE United States; 3 Ohia Advisors Boston, MA United States; 4 Isat advisors District of Columbia, MD United States

**Keywords:** health disparities, COVID-19, healthcare professional education, quality improvement, cultural sensitivity

## Abstract

**Background:**

The COVID-19 pandemic has underscored the need for targeted interventions to address health care disparities among specific health care professionals and mitigate the impact of the virus. In response, we developed a comprehensive statewide educational program protocol focused on subject areas of health equity, cultural sensitivity, infection prevention and control (IPC), and quality improvement (QI).

**Objective:**

The project aims to improve health care professionals’ knowledge and practice skills in the 4 subject areas, increase their comfort level in implementing health disparities-related QI projects, and facilitate the successful completion of QI projects addressing COVID-19 health disparities within their practice settings.

**Methods:**

The Reach, Effectiveness, Adoption, Implementation, and Maintenance (RE-AIM) framework was used in the planning and evaluation of this innovative educational program, which combines the Extension for Community Healthcare Outcomes (ECHO) learning model with one-on-one QI coaching. Participants engage in virtual interactive sessions led by experts and consultants, covering didactic presentations, case discussions, COVID-19 updates, and assessments. QI and health equity coaches provide guidance on developing QI projects targeting COVID-19 and other health disparities. Evaluation surveys are used for baseline, midpoint, and end-of-program assessment for self-reported comfort levels with knowledge and practice-based learning competencies in all 4 subject areas and health disparities-related QI project implementation. The Wilcoxon rank-sum test and Cochran-Armitage trend test will be used to compare pre- and postsurvey responses. Data from semistructured qualitative interviews, which capture insights into participants’ application of ECHO training, will be analyzed using an inductive content analysis approach.

**Results:**

A total of 50 ECHO sessions were held between November 2021 and May 2024. Overall, 510 participants attended at least one ECHO session, resulting in 3316 teaching encounters. The pre- and postsurvey data will be analyzed to study project impact and will be ready for publication in June 2026.

**Conclusions:**

By using implementation science methods, an innovative and comprehensive educational protocol was developed that integrates the training curriculum, evaluation metrics, and coaching support, allowing for the translation of the training into actionable community projects focused on addressing health disparities. This model has shown initial promise in terms of feasibility and uptake. Further studies are needed to evaluate the long-term effectiveness of these QI projects in reducing COVID-19 disparities.

**International Registered Report Identifier (IRRID):**

DERR1-10.2196/60901

## Introduction

During the early stages of the COVID-19 pandemic, health care workers in the United States had to quickly familiarize themselves with new guidance related to prevention, diagnosis, treatment, testing, and minimizing the spread of the virus [1]. Preventive strategies included nonpharmaceutical interventions such as mask-wearing, social distancing, and social isolation [1,2]. Despite these concerted efforts to contain the virus, COVID-19 spread and disproportionately impacted marginalized communities. African Americans, Native Americans, and Latinos were overrepresented among cases and deaths from COVID-19 [3,4]. Existing disparities in underlying conditions, known to be associated with COVID-19 mortality, including hypertension, cardiovascular disease, kidney disease, and diabetes, augmented the disproportionate hospitalization and deaths experienced by historically marginalized groups, including communities of color [5]. A meta-analysis of 72 studies (75% of which were from the United States) found that prevalence ratios of COVID-19 were much higher in African American individuals, Hispanic individuals, and those of other races (1.79, 1.78, and 1.43, respectively) than in White individuals (0.70). Higher burdens were also found in hospitalization and mortality ratios for African American individuals, Hispanic individuals, and those of other races than in White individuals [5,6]. Another study examining SARS-CoV-2–associated deaths reported that Latinx American individuals, African American individuals, American Indian individuals, and Alaska Native individuals accounted for approximately 75% of COVID-19 deaths in persons aged less than 21 years [7].

Disparities extended to COVID-19 testing, vaccine implementation, and treatment across various groups and settings [6,8,9]. Racially minoritized and low-income community members were less likely to be tested due to the possibility of lost wages, stigma, and mistrust. A total of 9 rural counties had significantly lower vaccine access and coverage than urban counties [6]. Safety and efficacy concerns made women and African Americans more likely to refuse vaccination [10]. Implicit bias among health care workers resulted in substandard care for people of color and prevented them from proactively seeking testing or timely treatment [6]. The pandemic highlighted the need for health care systems and professionals to recognize, acknowledge, and address health disparities.

Health care professionals who serve marginalized communities face many difficulties, including personal isolation, professional stagnation, excessive workload, and lack of access to consultation and continuing medical education [11]. The University of New Mexico’s Project Extension for Community Health Outcomes (ECHO) is an evidence-based telehealth intervention designed as a multidisciplinary virtual team-based educational program to overcome limitations in access to health care for vulnerable populations and individuals in rural areas [11,12]. Participation in Project ECHO improved provider knowledge, self-efficacy, and outcomes in patients with HIV [13], chronic pain [12], and substance use disorder [14].

Our “COVID-19 Disparities ECHO Project” seeks to reduce health disparities among high-risk and underserved populations in Nebraska through a multidisciplinary approach. As a longitudinal multilevel project, the Reach, Effectiveness, Adoption, Implementation, and Maintenance (RE-AIM) framework is being used to assess the dimensions of the project’s reach, efficacy, adoption, implementation, and maintenance. The 19-month program assists health care workers in identifying COVID-19 health disparities, developing targeted strategies to mitigate them by designing and implementing quality improvement (QI) projects, and promoting health equity and cultural sensitivity within their practice settings. There is a paucity of literature demonstrating effective interventions to reduce health disparities among underserved populations. This study protocol provides an in-depth description of the COVID-19 Disparities ECHO Project. It also highlights the rationale for an innovative educational approach that coalesces health equity and cultural sensitivity training with infection prevention and control (IPC) education and QI coaching in addressing COVID-19–related health disparities. Using the Standards for Quality Improvement Reporting Excellence (SQUIRE 2.0) [15] guidelines for QI publication, we describe the study overview, rationale for the study, specific aims, interventions, process and outcome measures, methods for qualitative and quantitative data analyses, ethical considerations, anticipated findings, potential impact, limitations, and future direction.

## Methods

### Overview of the COVID-19 Disparities ECHO Project

The COVID-19 Disparities ECHO Project is funded by the Nebraska Department of Health and Human Services through a Centers for Disease Control and Prevention (CDC) grant. The logic model assists with the planning, implementation, management, evaluation, and reporting of the COVID-19 Disparities ECHO Program (Textbox 1).

**Logic model describing the use of the logic model to characterize the inputs leading to the main impact of the Extension for Community Healthcare Outcomes program.**

**Inputs**

- Recruit subject matter experts and consultants.
- Faculty expertise and training resources in four subject areas:
- Quality improvement
- Health EquityA10357
- Cultural sensitivity
- Infection prevention and control
- QI tools to support participants’ projects

**Activities**

- Develop content and curricula for each of the 4 subject areas.
- Build and deliver an integrative curriculum.
- Enroll health care workers.
- Conduct periodic assessments of participants’ capability to inform the curriculum and coaching.
- Assist trainees with choosing, implementing, and completing quality improvement projects.

**Outcomes**

- Improve participants’ reported confidence in their training in the 4 subject areas.
- Improve participants’ perceived relevance of content from each of the 4 subject areas to their daily work.
- Improve participants’ reported comfort with core aspects of infection prevention and control related to COVID-19.
- Improve participants’ reported comfort with applying quality improvement principles and tools to their daily work in COVID-19.
- Improve participants’ reported comfort with applying health equity frameworks and tools to understand why some populations have been traditionally underserved and are at a higher risk for COVID-19.
- Improve participants’ reported comfort with using core principles of cultural sensitivity in their communications with populations that are traditionally underserved and at a higher risk for COVID-19.
- Improved state health department capacity and services to prevent and control COVID-19 infection (or transmission) among populations at high risk and underserved, setting the foundation to address future responses.

**Impact**

- To eliminate health disparities and improve the well-being of all Nebraskans.

The 19-month program consists of live virtual sessions held twice a month, each lasting 90 minutes. The 38 integrative sessions include 14 presentations on IPC and post–COVID-19 condition, 13 on cultural sensitivity principles, 20 on health equity, and 19 on QI. The presentation topics are adjusted based on the identified needs of the participants and the challenges they face with their QI projects. Participants who attend the sessions regularly and implement a QI project are eligible to receive a certificate of completion (“Health Equity and Quality Management Champion”) at the end of the program.

The course materials for the sessions are designed by the project directors and subject matter experts (SMEs) in infectious diseases, QI, and health equity, in partnership with our institution's Center for Continuing Education. The sessions are delivered twice monthly for 90 minutes via videoconferencing. These sessions include short didactic presentations with participant engagement, facilitated by interdisciplinary experts and guest faculty on featured topics. Case studies are used to help participants integrate and apply content from multiple subject areas. COVID-19 updates are also provided to assist learners in implementing best practices related to their health care settings.

Continuing education credits are available for physicians, advanced practice providers, nurses, social workers, and case managers at no cost through our institution’s Continuing Education offices. The available credits include the AMA (American Medical Association) PRA Category 1 Credit(s), American Nurses Credentialing Center (ANCC) contact hours, Association of Social Work Boards (ASWB) Approved Continuing Education (ACE) credits, and Pre-Approved Continuing Education (PACE) credits from the Commission for Case Manager Certification.

### Project Aims and Objective

The project aims to reduce COVID-19–related health disparities in Nebraska by educating and coaching health care workers in the following four subject areas: IPC, QI, Health Equity, and Cultural Sensitivity. Individual session objectives are developed by SMEs on the topics being discussed and are consistent with the program’s overall objectives, as outlined in Table 1. Coaching by QI and health equity experts are made available to the participants to assist them in implementing QI programs addressing COVID-19 disparities in their health care settings.

**Table 1:** The COVID-19 Disparities Extension for Community Healthcare Outcomes Project objectives.

Objectives	Description of objectives
Objective 1	Describe the principles of infection prevention and control and the skills required to implement these in their facilities, including challenges related to infrastructure, risk assessment, testing, contact tracing, vaccination, cleaning and disinfection, PPE^a^ usage, hand hygiene, quarantine and isolation, outbreak response, and the long-term complications and management of COVID-19 infection.
Objective 2	Recognize the role of relevant data collection and analyses in better understanding where and how to intervene to improve challenges related to infection prevention and control (eg, vaccine hesitancy, the reluctance to test, etc) for patients who are at higher risk and historically underserved, including communities of color and people living in rural communities.
Objective 3	Design, implement, and learn from changes to infection prevention and control practices in their facilities using the knowledge and skills gained during training to address inequities in COVID-19 prevention, diagnosis, and treatment in their communities.

^a^PPE: personal protective equipment.

The primary outcome measures for the study include a change in self-reported knowledge and practice-based competencies in all 4 subject areas and comfort level in implementing QI projects focused on reducing health disparities. Successful completion of QI projects is also monitored, in addition to self-reported comfort with QI project implementation. The process measures to track the successful implementation of projects include session attendance, participant retention, and initiation of new QI projects.

### Project Design and Implementation

#### Identifying an Implementation Strategy

The COVID-19 Disparity ECHO Project design was guided by the Consolidated Framework for Implementation Research (CFIR) and the Expert Recommendations for Implementing Change (ERIC, Detlev Offenbach) matching tool [16] to identify the implementation strategies necessary for creating a learning collaborative among program participants. One of these strategies is the creation of networks for communication to allow access to knowledge and information concerning COVID-19 disparities [16].

For COVID-19 Disparity ECHO Project to effectively educate health care professionals in COVID-19 prevention and disease management, it must translate into meaningful outcomes. As such, the RE-AIM framework was used in the planning and evaluation of the program [17]. RE-AIM evaluates interventions to optimize external and internal validity by addressing necessary measures for dissemination [18].

#### Reach

Reach is defined as the absolute number, proportion, and representativeness of individuals willing to participate in the ECHO program from the 93 counties and 19 local health departments in Nebraska.

#### Effectiveness

Effectiveness is the impact of an intervention on its outcomes. This measure will be evaluated using a mixed-mode approach. Surveys will be used to quantify the change in participants’ confidence skills and their intent to apply knowledge gained from the ECHO project. Qualitative interviews will be used to gain in-depth perceptions of the relevance of the ECHO topic areas and their experiences of applying ECHO topics in professional settings using a QI approach.

#### Adoption

Adoption is the representativeness of settings and intervention agents willing to initiate a program. QI coaching is provided to support QI projects designed by participants.

#### Implementation

Implementation refers to the intervention agents’ fidelity to the various elements of an intervention’s protocol. The Center for Continuing Education monitors the credibility of each session.

#### Maintenance

Maintenance is the extent to which a program or policy becomes institutionalized or part of routine organizational practices and policies. At the individual level, it is defined as the long-term effects of a program on outcomes 6 or more months after the most recent intervention contact. The program cultivates health equity and QI champions who will be equipped at the county, institutional, and regional levels to serve as local resources on these topics.

### Participant Recruitment

Nebraska is a largely rural state with many communities (66 of the 93 counties) defined as “medically underserved” [19,20]. To purposefully recruit participants across different health care organizations, the project team uses diversified recruitment techniques to reach organizations and individual health care workers. This includes announcing the training opportunity in various statewide health care facilities and local health department calls, sending emails to known distribution lists, and contacting health care personnel via telephone when the numbers are available. In addition, SMEs use word-of-mouth to recruit health care workers at affiliate organizations.

Participants from health care organizations whose representatives are enrolled in the project are eligible to receive the following benefits: (1) one-on-one coaching on QI and health equity, provided monthly or as requested, to develop and implement approved QI projects, (2) expense reimbursement of up to US $2000 per facility for costs related to their approved QI project, and (3) a certificate of completion from the COVID-19 Disparity ECHO Project recognizing their achievement as Health Equity and Quality Management Champion if they regularly participate in the training sessions and completed a QI project.

### Curriculum Development

The University of Nebraska Medical Center (UNMC) COVID-19 Disparities ECHO Project team faculty consists of UNMC SMEs who are divided into three teams, each responsible for developing and presenting content in the following subject areas: (1) IPC, (2) QI, and (3) Health Equity and Cultural Sensitivity. Three project directors collaborated with two project consultants to develop a curriculum that balanced and integrated content from all subject areas, focusing on building participants’ knowledge of how these subject areas inform and enrich one another. The project consultants served as experts and coaches in their respective areas of expertise and coordinated the contributions of other SMEs within their assigned faculty teams. The curriculum development is designed to be flexible, with a general outline prepared at the beginning of the project. Specific objectives of presentations and their sequence are adjusted based on periodic input from faculty and learners’ needs. One of the project consultants serves as the health equity and cultural sensitivity coach, while the other serves as the QI coach for the learners. Both are available to provide one-on-one assistance to learners upon request, outside of regular sessions.

### Structuring of ECHO Sessions

The sessions are conducted on Zoom (Zoom Video Communications Inc) [21]. Learners receive an email before each session containing the Zoom login information, a description of the activity, educational objectives, and requirements for successful completion to be eligible for continuing education credit for that particular session. Each session starts with a welcome message, disclosures, and an opening poll, followed by one or two short didactic sessions. A case study/discussion, COVID-19 updates, and a closing poll follow the didactic sessions. The details of these activities are outlined in Table 2. Each ECHO session is recorded, archived, and made available for viewing upon request after each session.

**Table 2:** General outlines of the COVID-19 Disparities ECHO^a^ Project sessions.

Time allocated	Session activity	Description of activity	ECHO team leading the activity
10 minutes	Introduction and disclosures	The sessions begin with introducing the topic and disclosing the presenter’s conflicts of interest.	Project manager
10 minutes	Opening poll	Participants are asked to choose one of following four responses regarding their experience in the previous session: (1) I learned something new that I have applied in my work setting, (2) I learned something new that I am planning to apply in my work setting, (3) I was reminded of how I can apply the information I already know in my work setting, and (4) I do not plan to apply this content to my work setting.	Quality improvement team
30 minutes	Didactics	Each session can feature up to two didactics covering one or two of the project’s four subject areas. SMEs^b^ and guest presenters deliver a short presentation for each didactic. SMEs develop interactive presentations that engage participants through discussion questions, polls, and interactive examples. Topics are selected and paired intentionally to highlight how subject areas contribute to and enrich one another.	SMEs on quality improvement, cultural sensitivity/health equity, and infection prevention or control
20 minutes	Case study or discussion	Each session includes a case study or discussion section which draws on subject matter from the didactics. The project consultants develop these collaborative exercises in collaboration with SMEs to reinforce the connections between subject matter areas.	SMEs on quality improvement, cultural sensitivity/health equity, or infection prevention and control
10 minutes	COVID-19 updates	Updates on COVID-19 in Nebraska are shared, including newly reported cases, hospital admissions, test positivity rates, mortality rates, and vaccination rates for COVID-19.	SME on infection prevention and control
10 minutes	Closing poll	At the conclusion of each session, participants respond to a poll regarding the session’s content. Participants are asked to rate their confidence that they could apply that session’s content in their work setting with one of the following four responses: Extremely, Moderately, Slightly, and Not at all.	Quality improvement team and project manager

^a^ECHO: Extension for Community Healthcare Outcomes.

^b^SME: subject matter expert.

### Facilitating Quality Improvement Projects

The project team envisioned QI as both a subject matter area and a methodology to support participants’ learning across all 4 subject matter areas. Year 1 of the project is focused on learning about the critical components of practical QI projects through didactic presentations, case studies, and interactive discussion exercises. During year 2, participants can receive coaching support for a QI project that addresses COVID-19 disparities in their facilities. QI content in year 2 sessions is designed to reinforce and support participation in these QI projects.

QI projects must address one or more areas of need relevant to COVID-19 and health equity (or cultural sensitivity) in their communities (Textbox 2). The learners submit QI Project proposals through the REDCap (Research Electronic Data Capture; Vanderbilt University) software [22]. The QI and health equity coaches review the proposals before acceptance. Accepted projects are assessed by the QI faculty team using the Quality Improvement Knowledge Application Tool Revised (QIKAT-R) [23]. The QIKAT-R assesses an individual’s ability to decipher a quality problem within a complex system and propose an initiative for improvement.

**The COVID-19 Disparities Extension for Community Healthcare Outcomes (ECHO) project’s suggestions of health equity and COVID-19 infectious disease prevention and control topic areas for quality improvement projects.**

**COVID-19 topic areas (participants’ quality projects integrate one topic area from the suggestions of COVID-19 and health equity topics each)**

- Vaccination and vaccine support
- Testing
- Contact tracing
- Case investigation
- Quarantine and isolation
- Preventive care and disease management
- The long-term impact of COVID-19
- Personal protective equipment (PPE)
- Non–health care services related to COVID-19 (ie, transportation and food assistance)
- Evidence-based policies or systems (ie, risk assessment, screening, and visitation)
- Environmental strategies (ie, cleaning or disinfection)
- Navigation and support services to address COVID-19 risk and prevention
- Communications about COVID-19 risk and prevention
- Plans for countermeasures and adaption services

**Health equity topic areas (participants’ quality projects integrate one topic area from the suggestions of COVID-19 and health equity topics each)**

- Racial/ethnic identity
- Gender identity
- Sexual orientation
- Neighborhood/physical environment (eg, air/water quality, housing, and violence)
- Economic stability (eg, employment and poverty)
- Citizenship/immigration status
- Education access, quality, and literacy level
- Health care access, quality, and health literacy level
- Social and community context (eg, discrimination, family support, and community support)
- Cultural sensitivity (eg, religious sensitivity)

### Data Collection

Monitoring and evaluating outcomes are fundamental to the ECHO model. The COVID-19 Disparities ECHO project used a mixed-mode approach to collect data for planning and assessing continuous learning guided by the RE-AIM Framework.

### Quantitative Data

#### Session Attendance

Participant attendance and retention rates are collected using program registration, session registration, and continuing education claim data.

#### Session Polls

Participants are asked to respond to two polls—one at the conclusion of each session and another at the beginning of the following session—regarding their satisfaction with the didactic and case presentations, self-reported changes in knowledge, and the likelihood of implementing this knowledge (outlined in Table 2 for the session details).

#### Evaluation Surveys

Evaluation surveys are used for baseline, midpoint, and end-of-program assessment to gauge the program’s impact on learning and comfort in implementing that learning in professional settings. While responses are submitted anonymously to protect participant privacy, information on participants’ roles, workplaces, and demographic characteristics are collected to assess any differences in program outcomes across these factors. The evaluation surveys included questions on participants’ confidence in training received to date in each subject area, perceived relevance of each subject area to their daily work, comfort with a set of knowledge and practice-based competencies in each subject area, and comfort with data collection/analysis and change implementation.

#### Quality Improvement Project Participation

The number of projects proposed, started, and completed will be counted and analyzed for COVID-19 and the health equity they addressed. The QIKAT-R tool is used to evaluate QI projects on submission, identify areas for improvement, and structure coaching support for participants to improve their ability to charter practical QI projects.

### Qualitative Interviews

Qualitative interviews will be used to gather additional data on participants’ experience implementing program learning in their workplaces. It consists of structured 30-minute interviews using a standardized protocol (Multimedia Appendix 1) with participants engaged in program sessions regardless of whether they chose to submit a formal QI project. Discussion during interviews focuses on participants’ motivations for joining the program, previous experience with QI projects, experience in the program, and what changes they had been able to make or planned to make in their workplaces in each of the 4 subject areas.

### Data Analyses

#### Quantitative Data Analyses

Descriptive statistics, including means, SD, medians, ranges, IQR, counts, and percentages, will be used to summarize pre- and postsurvey responses. Fisher exact test will be applied to compare the distribution of roles and workplaces between pre- and postsurvey participants. To compare pre and post responses, two methods will be used. First, the Wilcoxon rank-sum test will examine differences between the pre- and postsurvey groups across domains and subdomains. In addition, the Cochran-Armitage trend test will be used to compare pre and post responses, treating the data as ordinal categories. Comparisons of domain data stratified by role or workplace will also be performed using the Wilcoxon rank-sum test. All statistical analyses will be conducted using SAS Version 9.4 (SAS Institute), with a *P* value of <.05 considered statistically significant.

#### Qualitative Data Analysis

The qualitative data from the interviews will be analyzed using an inductive content analysis approach by 2 individuals. The analysts will first familiarize themselves with the interview transcripts, and then independently code the data by identifying key segments related to participants’ motivations, previous experience with QI projects, program experiences, and workplace changes. These codes will be grouped into broader themes, such as barriers to change or motivational factors. These themes will be further compared and refined through discussion and input from a third individual not included in the analysis. The final themes will reflect the participants’ experiences and will be interpreted in relation to the project’s aims and objectives.

### Ethical Considerations

The study was determined to be “Not a Human Subject Research” (due to being a program evaluation for a QI project) by the UNMC institutional review board (0561-23-EX). Formal “Informed Consent” is not required and does not apply to this study as it is a program evaluation for a QI project. All project data are maintained in secured online folders with access limited to the project team members. Only aggregated and/or deidentified data will be shared or reported, and participants will not be identified. No compensation is provided directly to the project participants. However, participants are eligible for claiming free educational credits for their participation in educational sessions. Furthermore, their organization is eligible for expense reimbursement for implementing approved QI projects as part of the training program.

## Results

Between November 2021 and May 2023, a total of 38 ECHO sessions (2 sessions per month) were delivered. QI Coaching was provided to 23 participants (representing 15 organizations), who mentioned their interest in initiating a project to address health disparities. A total of 12 additional 60-minute ECHO sessions were organized on a monthly basis between June 2023 to May 2024 to provide ongoing guidance and support for participants already working on their QI projects and to encourage more participants to initiate their own QI projects. That resulted in 22 participants (including 6 new, who did not work on a QI project earlier) reaching out with interest and receiving coaching to work on their QI projects.

Overall, 510 participants attended at least one ECHO session between November 2021 and May 2024 resulting in 3316 teaching encounters. Data related to participants’ self-reported comfort level with a core set of knowledge and practice-based competencies in all four subject areas, self-reported comfort with data collection, data analysis and change implementation, attendees’ interviews, and QI project implementation will be analyzed to assess the impact of this project and expected to be ready for publication in June 2026.

## Discussion

### Overview

This well-attended innovative educational project is expected to improve participants’ self-reported knowledge and practice-based competencies in all four subject areas (IPC, QI, health equity, and cultural sensitivity). Participants are also expected to report an increase in their comfort level with implementing QI projects designed to reduce health disparities. Furthermore, QI project participation by the learners is anticipated to result in the completion of a wide range of health equity projects. Qualitative interviews of the participants are expected to provide insights into the factors associated with successful program implementation and the challenges linked to planning and implementing projects focused on reducing health disparities.

There is a critical need to address COVID-19 and other health disparities in rural states with primarily medically underserved and minoritized communities. Like other Midwest states, Nebraska has shown higher COVID-19 mortality rates among Blacks compared with Whites [24,25]. Midwestern states have a history of well-documented racial disparities in education, incarceration, employment, income, health, medical care, homeownership, voting access, wages, and numerous other socioeconomic factors [25,26]. Therefore, beyond understanding IPC principles, health care professionals, who are often the first point of contact for minoritized individuals, require an in-depth understanding of these complex drivers of COVID-19 inequities. Health education must be designed to improve how care is provided in health care settings to cultivate a more profound knowledge of the structural inequities that drive health disparities. The findings from this study are expected not only to highlight the knowledge gap of health care workers related to health equity and cultural sensitivity principles but also to provide evidence that a well-designed educational program can mitigate those gaps.

The ECHO model’s use of case-based presentations, supplemented by short didactic presentations by SMEs, has been shown to improve the skills, knowledge, and ability to provide treatment by providers in underserved communities [27]. This model, by its design, addresses one of the most significant health care disparities in predominantly rural states (such as Nebraska), which relates to the lack of access to SMEs in rural communities. Choosing the ECHO model for our training allowed us to connect health care workers from across the state to SMEs in all four subject areas: IPC, health equity, cultural sensitivity, and QI. To address health disparities, health care workers need to have a basic understanding of existing health disparities in their practice areas and the underlying factors responsible for those disparities. SMEs in the area of health equity and cultural sensitivity prepared specific content throughout the training to explain these concepts. They also highlighted the need for health care professionals to gather, review, and analyze relevant data that is essential to gain deeper understanding of health disparities within their own practice settings and communities. QI SMEs provided one-on-one and group coaching, not only on how the participants can analyze the data for health disparities but also on how to set priorities, plan interventions, anticipate challenges, monitor progress, overcome barriers, and engage with relevant stakeholders to address the root causes of targeted health disparities.

Studies conducted before the COVID-19 pandemic highlighted the urgent need for interventions targeting factors that contribute to health care professionals’ role in perpetuating health care disparities, such as racial and ethnic biases [28]. Attention to these subjects heightened following the disproportionate burden of the COVID-19 pandemic on minoritized populations. The case-based learning and discussion allow health care workers to understand how health equity and cultural sensitivity concepts directly apply to patient care. Therefore, the ECHO model is well-suited for training health care workers to reduce health disparities. Case-based learning during ECHO sessions has usually been used to discuss individual patient care during educational sessions. We took a modified approach and expanded case-based learning section to include discussions on nonclinical scenarios or focused questions that address broader problems in health care systems relevant to addressing COVID-19 and other health disparities [11,12].

More recently, The Joint Commission has revised the Leadership Standard for ambulatory care organizations, behavioral health care and human services organizations, critical access hospitals, and hospitals to address health care disparities as a quality and safety priority [29]. The COVID-19 Disparity ECHO Project curriculum provides an ideal outline for future educational projects focused on training health care leaders and frontline staff on taking a QI approach to identify and reduce various health care disparities.

The ECHO model has limitations. Traditional ECHO projects are best suited for providing educational tools for direct patient care rather than addressing the organizational and systemic changes needed to tackle COVID-19 disparities. Studies have shown that addressing health disparities requires the development of specific, measurable objectives and implementing strategies that will successfully address those inequities [30]. QI approaches are well-suited for addressing disparities because they offer strategies to target modifiable aspects of care delivery and a method for tailoring or changing an intervention. Integrating QI principles into our curriculum, including the novel approach of one-on-one coaching, assists health care workers in designing and implementing QI projects to reduce health care disparities in their communities. Adding QI and health equity coaches to the traditional ECHO model assists health care workers in applying the knowledge gained through training to their practice. We will gain more insights into this by monitoring the QI projects planned and completed by the participants enrolled in the training program.

While the new and innovative educational design combining the ECHO model with QI coaching and the participation of over 500 learners are strengths of our study, it also has some limitations. This is a single-center study limited to Nebraska and does not include a control group because the project was funded to offer this educational program to all health care professionals in the state. Furthermore, the actual impact of this project on the launch of new QI projects in the state for addressing COVID-19 and other health disparities is likely going to be underestimated, as many of the participants will continue to apply the lessons learned from this educational program for years to come. Another limitation is our inability to monitor participants’ initiated QI projects for effectiveness in reducing COVID-19 health disparities because of the short follow-up timeframe. However, all QI projects proposed by the participants must be submitted to the project team for review and approval. Participants receive feedback for improvement and structured coaching support to ensure the successful completion of their QI projects, which is tracked as one of the outcome measures of this study. Although we are not able to follow up on the effectiveness of these QI projects in reducing health disparities, participants have received education, coaching, and tools to assist them in monitoring their own program effectiveness. Future studies will need to be conducted to evaluate the long-term effectiveness of these educational project-driven QI projects in reducing COVID-19 health disparities.

Findings from this project will be shared with participants, state and local stakeholders, and the general audience through our website and scientific publications. Future work should focus on evaluating the impact of ECHO training on patient-level outcomes including morbidity and mortality related to COVID-19 and other infectious diseases. In addition, this project model can be adapted in the future to train health care professionals in addressing health disparities related to various other infectious and noninfectious diseases, especially in resource-limited settings.

### Conclusions

By using implementation science methods, an innovative and comprehensive educational protocol was developed for health care workers in Nebraska that integrates the training curriculum, evaluation metrics, and coaching support, allowing the translation of the training into actionable health care/community projects focused on addressing health disparities. This model has shown initial promise in terms of feasibility and uptake. Further studies to evaluate the long-term effectiveness of these QI projects in reducing COVID-19 disparities are needed.

## Appendix

Multimedia Appendix 1 Qualitative interview of the UNMC (University of Nebraska Medical Center) ECHO (Extension for Community Healthcare Outcomes) program.

## Abbreviations

ACE: Approved Continuing Education
AMA: American Medical Association
ANCC: American Nurses Credentialing Center
ASWB: Association of Social Work Boards
CDC: Centers for Disease Control and Prevention
CFIR: Consolidated Framework for Implementation Research
ECHO: Extension for Community Healthcare Outcomes
IPC: infection prevention and control
PACE: Pre-Approved Continuing Education
QI: quality improvement
QIKAT-R: Quality Improvement Knowledge Application Tool Revised
RE-AIM: Reach, Effectiveness, Adoption, Implementation, and Maintenance
REDCap: Research Electronic Data Capture
SME: subject matter expert
SQUIRE: Standards for Quality Improvement Reporting Excellence
UNMC: University of Nebraska Medical Center

## Data Availability

The datasets generated during and/or analyzed during this study are available from the corresponding author upon reasonable request.
