# Supplementary material for: Providing Education and Training to Health Care Professionals to Address COVID-19 Health Disparities: Protocol for Implementation Project Using Reach, Effectiveness, Adoption, Implementation, and Maintenance Framework
Source: JMIR Res Protoc. 2025 May 16;14:e60901. doi: 10.2196/60901 (PMC12125559; doi:10.2196/60901)
Supplement: Multimedia Appendix 1 [file resprot_v14i1e60901_app1.pdf]

Appendix A: Qualitative Interview Guide for COVID-19 Disparities ECHO Project

Introduction

1. What motivated you to join this ECHO?
2. What past experiences have you had with quality improvement projects before joining this course?

Health Equity

3. Can you share an example of an idea for change relevant to health equity that you have been able to implement?
    a. If not, can you share an example of an idea you hope to implement?
        i. If not, what else could the program have focused on to help you implement ideas in this area?

Infection Prevention and Control

4. Can you share an example of an idea for change relevant to infection prevention and control, either for COVID-19 or other infectious diseases, that you have been able to implement?
    b. If not, can you share an example of an idea you hope to implement?
        i. If not, what else could the program have focused on to help you implement ideas in this area?

Cultural Sensitivity

5. Can you share an example of an idea for change relevant to cultural sensitivity that you have been able to implement?
    c. If not, can you share an example of an idea you hope to implement?
        i. If not, what else could the program have focused on to help you implement ideas in this area?

Quality Improvement

6. Can you share an example of an idea for change relevant to quality improvement that you have been able to implement?
    d. If not, can you share an example of an idea that you hope to implement?
        i. If not, what else could the program have focused on to help you implement ideas in this area?
7. Can you share an example of how participating in this ECHO impacted your ability to assess the impact of changes within your organization?
    e. If not, what else could the program have done to help you in this area?

Closing

8. Overall, what aspect(s) of this ECHO have been most helpful to you?
9. Overall, what aspect(s) of this ECHO have been most challenging for you?
